# Liver Frailty Index for Prediction of Short-Term Rehospitalization in Patients with Liver Cirrhosis

**DOI:** 10.3390/diagnostics12051069

**Published:** 2022-04-24

**Authors:** Leonard Kaps, Lejla Lukac, Maurice Michel, Wolfgang Maximilian Kremer, Max Hilscher, Simon Johannes Gairing, Peter R. Galle, Jörn M. Schattenberg, Marcus-Alexander Wörns, Michael Nagel, Christian Labenz

**Affiliations:** 1 Department of Internal Medicine I, University Medical Centre, Johannes Gutenberg-University, 55131 Mainz, Germany; lukac0102@gmail.com (L.L.); maurice.michel@unimedizin-mainz.de (M.M.); maximilian.kremer@unimedizin-mainz.de (W.M.K.); max.hilscher@klinikumdo.de (M.H.); simonjohannes.gairing@unimedizin-mainz.de (S.J.G.); peter.galle@unimedizin-mainz.de (P.R.G.); joern.schattenberg@unimedizin-mainz.de (J.M.S.); marcus.alexander.woerns@klinikumdo.de (M.-A.W.); michael.nagel@klinikumdo.de (M.N.); 2 Cirrhosis Centre Mainz (CCM), University Medical Centre, Johannes Gutenberg-University, 55131 Mainz, Germany; 3 Department of Gastroenterology, Hematology, Oncology and Endocrinology, Klinikum Dortmund, 44137 Dortmund, Germany

**Keywords:** end-stage liver disease, complications of cirrhosis, point-of-care diagnostic, functional decline, extrahepatic features of chronic liver disease, sarcopenia

## Abstract

Background: Stratifying patients with liver cirrhosis for risk of rehospitalization is challenging with established scoring systems for chronic liver disease. Frailty captures the physical characteristics of patients with cirrhosis. Its value for predicting short-term rehospitalizations in hospitalized patients remains to be defined. Methods: Eighty-three non-electively hospitalized patients with liver cirrhosis were analyzed in this study. Frailty was assessed during the last 48 h of hospital stay with the liver frailty index (LFI). Patients were followed for 30-day rehospitalization. Results: In total, 26 (31%) patients were rehospitalized within 30 days. The median LFI was 4.5, and 43 (52%) patients were identified as frail. Rehospitalized patients had a significant higher LFI compared to patients without a rehospitalization within 30 days. In multivariable analysis, LFI as a metric variable (OR 2.36, *p* = 0.02) and lower platelet count (OR 0.98, *p* < 0.01) were independently associated with rehospitalization. LFI and its subtest chair stands had the best discriminative ability to predict rehospitalization, with AUROCs of 0.66 and 0.67, respectively. An LFI cut-off of >4.62 discriminated best between patients with and without elevated risk for rehospitalization within 30 days. Conclusions: Measures of frailty could be useful to identify patients at higher risk for short-term rehospitalization.

## 1. Introduction

Frailty represents a clinical syndrome complex including a decline in physical and physiological reserves, ultimately leading to vulnerability to adverse health outcomes [1,2]. In the past, frailty was a condition exclusively associated with aging and was considered more of a geriatric problem, whereas currently, its relevance is broadly accepted in chronic diseases [3].

Liver cirrhosis is the end-stage of almost every chronic liver disease. It is characterized by an excessive accumulation of scare tissue, which disrupts the liver’s delicate cellular architecture [4]. Patients with liver cirrhosis often present with a wide range of clinical symptoms, including muscle wasting and neurological disorders such as hepatic encephalopathy, which contributes to a decline in physical function [5,6]. Established scores for assessing liver disease severity, such as the Model for End-Stage Liver Disease (MELD), are based exclusively on serum parameters [7]. Although the MELD score is still considered as the gold standard for predicting mortality in patients with end-stage liver disease, it does not capture the extrahepatic features of cirrhosis, such as muscle wasting, malnutrition, and functional decline [8].

In recent years, the liver frailty index (LFI) has emerged as a valid and easy-to-use tool to assess frailty in patients with liver cirrhosis. LFI is based on three objective physical performance tests, including hand-grip strength, chair stands, and balance exercises, and has been shown to improve risk prediction for mortality in cirrhotic patients awaiting liver transplantation [9]. In addition, LFI predicts cirrhosis progression, excess mortality independent of underlying liver function, and unplanned hospitalizations in outpatients with compensated and decompensated cirrhosis [10]. While several tools exist to predict hospitalizations in outpatients with cirrhosis, predicting rehospitalizations in inpatients with cirrhosis remains complex. Tapper et al. demonstrated that measures of frailty such as the Braden scale were associated with length of stay or discharge of patients with cirrhosis to a rehabilitation facility [8]. However, data on the predictive ability of the LFI to predict rehospitalizations, especially from Europe, are lacking. 

This study aimed to evaluate the usefulness of the LFI for predicting short-term rehospitalization of hospitalized patients with liver cirrhosis.

## 2. Patients and Methods

### 2.1. Patients

**Figure 1:**
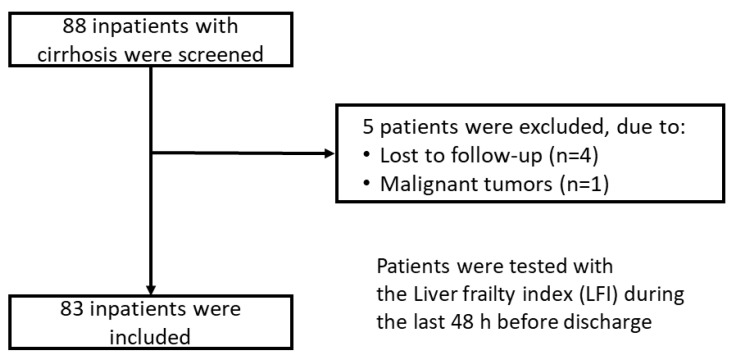
Consort flow chart of the study.

In total, 88 cirrhotic inpatients were prospectively recruited, and data were included into a database between September 2019 and December 2020 at the Cirrhosis Center Mainz of the University Medical Centre of the Johannes Gutenberg–University in Mainz (Germany). For this post hoc analysis (retrospective), five patients were excluded due to active malignancies or loss to follow-up (Figure 1). Finally, data of 83 patients were retrospectively analyzed. The primary endpoint of this study was an unplanned rehospitalization within 30 days from the time of hospital discharge. The incidence of rehospitalization for each patient was determined retrospectively by electronic chart review. 

Patient characteristics were recorded during the last 48 h of each hospital stay. Etiology of underlying liver disease was determined according to clinical, serological and histological findings together with anamnesis. Cirrhosis was diagnosed by an experienced hepatologist considering histology, typical appearance in ultrasound or radiological imaging, endoscopic features of portal hypertension, and medical history. History of decompensation and blood biochemistry were recorded in detail for each patient. MELD and Child-Pugh (CP) score were calculated to determine the severity of liver disease [7,11].

### 2.2. Liver Frailty Index

After recompensation, each patient was tested with the LFI as part of routine clinical practice during the last 48 h of their respective hospital stay. The LFI consists of the following three physical tests and was administered by a trained healthcare professional:

1. Hand grip strength: the average of three trials, measured on the patient’s dominant hand using a hand dynamometer.
2. Chair stands: measured as the number of seconds the patient needs to perform five chair stands with arms folded across the chest.
3. Balance testing: measured as the number of seconds the patient manages to balance in three positions (feet placed side-to-side, semi tandem, and tandem) for a maximum time of 10 s each.

LFI was calculated based on the results of the administered tests, applying the online available calculator at http://liverfrailtyindex.ucsf.edu (accessed during the study period September 2019–December 2020). Higher LFI values indicate a higher degree of physical functional impairment. Patients with an LFI value of ≥4.5 were considered frail [9].

### 2.3. Ethics

The study was conducted according to the ethical guidelines of the 1975 Declaration of Helsinki and its later amendments. The study was approved by the ethics committee of the Landesärztekammer Rhineland-Palatinate. Written informed consent was obtained from each participant.

### 2.4. Statistical Analysis

Data were analyzed using IBM SPSS Statistic Version 27.0 (Armonk, NY, USA: IBM Corp.) and GraphPad Prism Version 8.0.2 (GraphPad Software, San Diego, CA, USA).

Quantitative data are expressed as medians with interquartile ranges (IQR), and pairwise comparisons for quantitative variables were performed with an unpaired *t* test or with the Mann–Whitney U Test. Categorical variables are expressed as frequencies and percentages. For comparison of two or more patient groups, a chi-square test was applied. Correlation analyses were conducted using Spearman’s rank correlation.

To reliably identify factors being associated with 30-day rehospitalization, we conducted multivariable logistic regression models based on a stepwise variable selection procedure. To investigate the discriminate ability of the respective regression models, Harrell’s C-index was calculated. Additionally, we also conducted time-to-event analyses using Kaplan–Meier curves and Cox-regression analysis based on a stepwise variable selection procedure.

To investigate how the LFI and its subtests discriminate between patients with and without rehospitalization within 30 days, we calculated area under the curve of receiver operating characteristic (AUROC) curves and their respective 95% confidence intervals (95% CI). Thresholds for the LFI and its subtests were determined based on the Youden’s index (equal weighting of sensitivity and specificity).

Our complete data analysis is exploratory. Hence, no adjustments for multiple testing were performed. For all tests, we used a 0.05 level to define statistically relevant deviations from the respective null hypothesis. However, due to the large number of tests, *p* values should be interpreted with caution. 

## 3. Results

### 3.1. Patient Characteristics at Baseline

**Figure 2:**
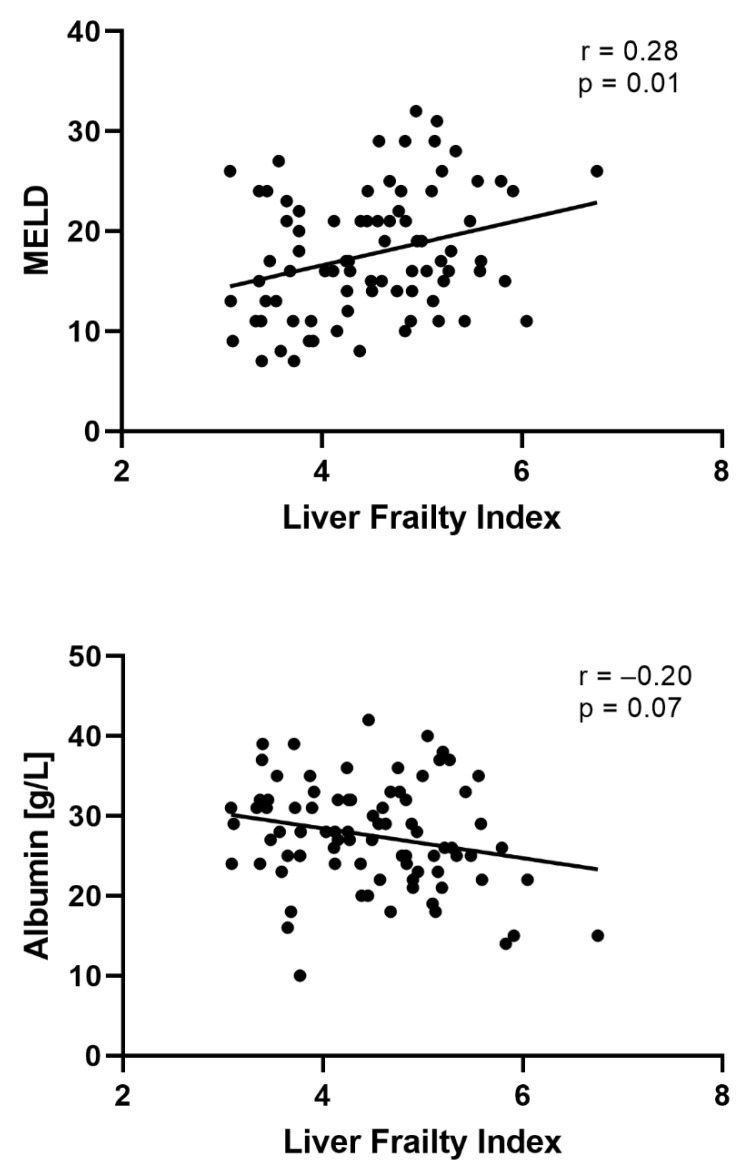

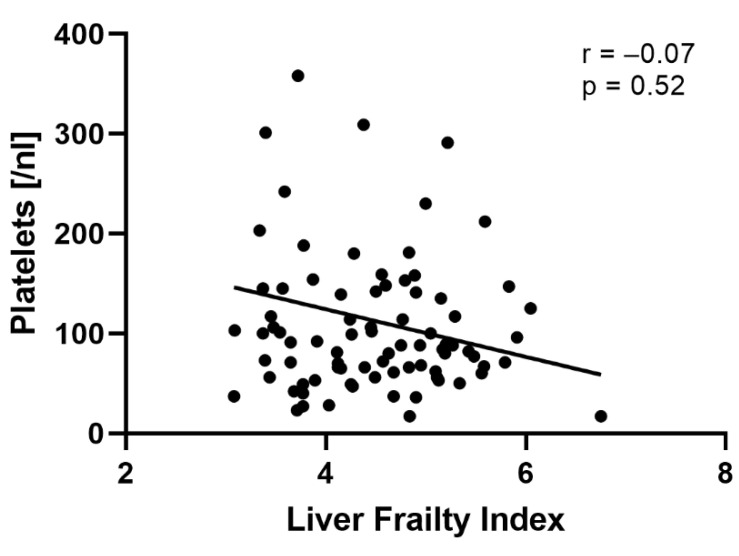
Correlation between MELD, albumin, platelets and the liver frailty index.

In total, data of 83 inpatients with liver cirrhosis were analyzed in this study. Sixty percent of the patients were male with a median age of 60 years (IQR 51; 67). The main etiology for liver cirrhosis was chronic alcohol consumption (66%). The median MELD score at study inclusion was 17 (IQR 13; 22), while 31% were categorized in Child-Pugh class C. In the total cohort, the median LFI was 4.5 (IQR 3.8; 5.1), which corresponded to 52% of the patients being classified as frail. The majority of patients had a history of ascites (83%), and 35% had a history of HE. Additional baseline characteristics of the entire cohort are displayed in Table 1. LFI only had a weak correlation with measures of liver function and portal hypertension such as MELD, albumin or platelets (Figure 2).

**Figure 3:**
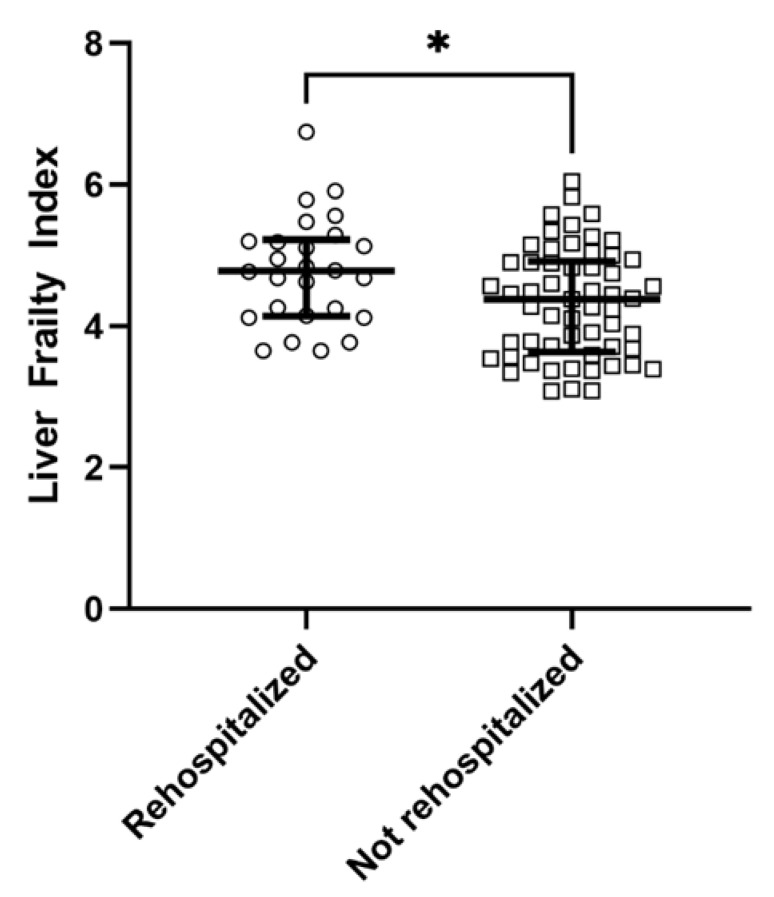
Median liver frailty index in patients with and without rehospitalization within 30 days from discharge (* *p* < 0.05).

In total, 26 (31%) patients were rehospitalized within 30 days, and no patient died without being rehospitalized. Volume overload was the most frequent (75%) diagnosis leading to rehospitalization. Rehospitalized patients differed significantly from patients without rehospitalization, for instance in terms of MELD score, history of ascites and LFI. The comparisons between both groups are displayed in Table 1. The comparison of the LFI between patients with and without rehospitalization is displayed in Figure 3.

Data are expressed as median with interquartile ranges (IQR) or as frequencies with percentages. HE, hepatic encephalopathy; LFI, liver frailty index; NAFLD, non-alcoholic fatty liver disease; SBP, spontaneous bacterial peritonitis; WBC, white blood cell count.

### 3.2. Factors Associated with Non-Elective Rehospitalization within 30 Days

A multivariable logistic regression model with a stepwise variable selection process was applied to assess risk factors for non-elective rehospitalization within 30 days. In a model including LFI as a metric variable, lower platelet counts (OR 0.98, *p* < 0.01) and higher LFI (OR 2.36, *p* = 0.02) were independently associated with rehospitalization within 30 days (Table 2). In a separate model, we included the LFI as a categorial variable (robust + prefrail vs. frail). Here, frailty, as defined by an LFI > 4.5, was not associated with a rehospitalization within 30 days (Table 2). The results of the regression model did not change when the MELDNa score was considered instead of the MELD score (*p* = 0.09 for MELDNa score).

To strengthen our findings, we repeated our analysis using a multivariable Cox-regression analysis with a stepwise variable selection process. In this model including LFI as a metric variable, lower platelet counts (HR 0.98, 95% CI 0.97–0.99, *p* = 0.01) and higher LFI (HR 1.65, 95% CI 1.07–2.55, *p* = 0.02) were independently associated with time to rehospitalization within 30 days (Supplementary Table S1).

### 3.3. LFI Predicts 30-Days Rehospitalization of Patients with Liver Cirrhosis

**Figure 4:**
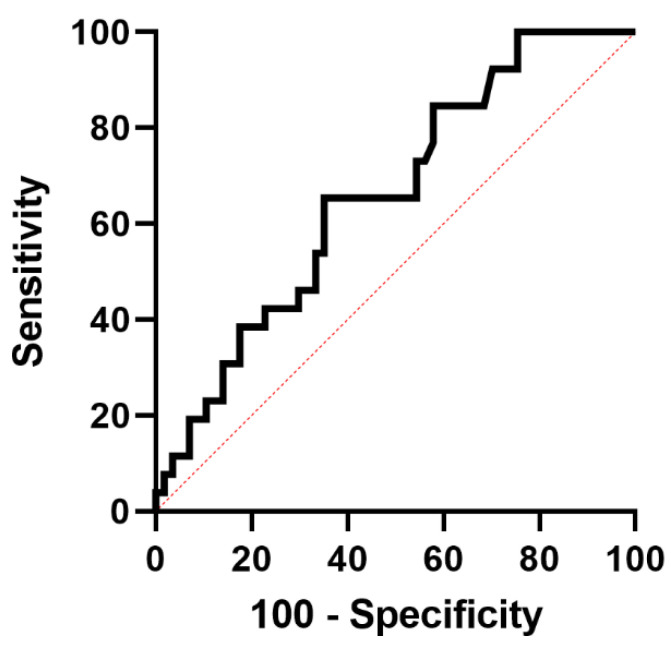
Receiver operating characteristic (ROC) curve for the liver frailty index (LFI) to predict 30-day rehospitalization in patients with liver cirrhosis.

**Figure 5:**
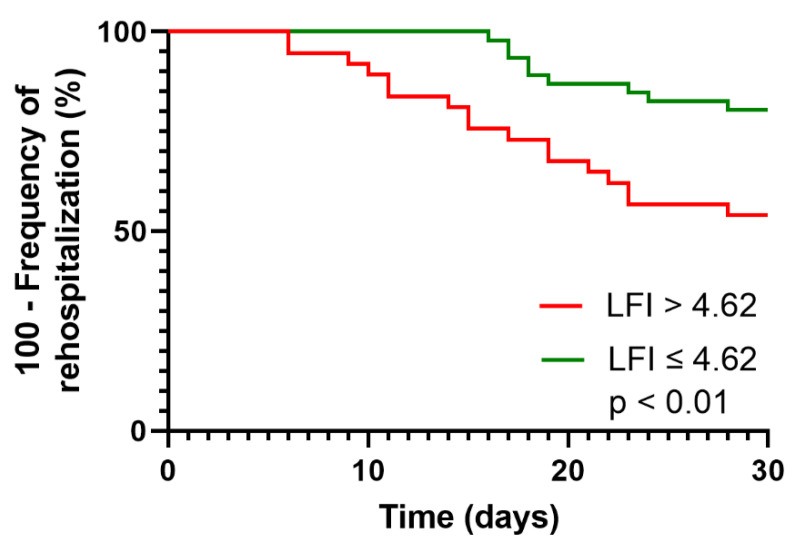
Kaplan–Meier curve displaying the time to rehospitalization in the total cohort stratified by an LFI cut-off of 4.62.

We conducted ROC analyses to assess the performance of the LFI and each of its subtests to predict rehospitalization within 30 days (Figure 4). AUROCs were numerically highest for the LFI (AUC 0.66, 95% CI 0.54–0.78) and its subtest chair stands (AUC 0.67, 95% CI 0.55–0.79), followed by the balance tests tandem stand (AUC 0.61; 95% CI 0.48–0.75) and semi-tandem stand (AUC 0.59, 95% CI 0.46–0.73), while side stand (AUC 0.53; 95% CI 0.40–0.67) and handgrip strength (AUC 0.58; 95% CI 0.44–0.71) had the lowest discriminative ability (Table 3). The ideal cut-off of the LFI to predict rehospitalization within 30 days was 4.62 according to the Youden’s Index. Using this cut-off, the respective sensitivity and specificity were 65% each. AUROCs and cut-offs of the LFI and each of its subtests are displayed in Table 3. Using the cut-off of 4.62 to stratify the patient cohort, patients with an LFI above the cut-off had a significantly higher rehospitalization rate in time-to-event analysis (Kaplan–Meier curve, Figure 5, *p* < 0.01). 

## 4. Discussion

Predicting rehospitalization of patients with liver cirrhosis is challenging in clinical practice. In this study, we found that higher LFI scores—a measure of frailty—were independently associated with a higher risk of 30-day rehospitalization in hospitalized patients with liver cirrhosis. Additionally, we demonstrated that the diagnostic accuracy to predict rehospitalization was comparable between LFI and its subtest chair stands, although the discriminative ability of these measures was only mediocre. These findings expand the growing body of evidence indicating the usefulness of frailty assessments for prediction of clinical outcomes in patients with liver cirrhosis.

Frailty is common in patients with liver cirrhosis and is associated with a higher mortality in outpatients irrespective of other cirrhosis-related complications [12]. Studies on outpatients with liver cirrhosis indicated a prevalence of frailty of up to 25% using the LFI for frailty assessment [12]. However, data on the prevalence of frailty in inpatients are scarce. A North American-based study found a frailty prevalence of 59% in hospitalized patients with liver cirrhosis [13]. Our current findings expand the literature as we investigated frailty of hospitalized patients with liver cirrhosis for the first time in Germany, using the LFI as robust measure. In line with the results presented by Serper et al., we found a similar high prevalence of frailty (51%) in our cohort. The high prevalence may be explained by a combination of preexisting frailty prior to hospitalization and acute illness-derived frailty caused by acute stressors that led to hospitalization. The high prevalence of frailty in our cohort is not fully reflected by established measures of liver function, e.g., the MELD score or albumin (Spearman’s rho ≤ 0.28). This emphasizes the fact that frailty is not restricted to hepatic function, which is measured by serum parameters and single clinical features, but which also captures relevant extrahepatic manifestation such as sarcopenia, malnutrition or cognitive function [5,14,15]. The identification of patients at high risk for hospitalization is pivotal to establish preventive measures. Wang et al. reported in a multicenter study that frail outpatients have an increased risk (hazard ratio (HR) 2.32) of unplanned hospitalizations [10]. In our current study, we were able to demonstrate that the LFI has the potential to help identify hospitalized patients at higher risk for short-term rehospitalization. This finding is in agreement with the United States-based multi-center study conducted by Serper et al. [13]. Here, the authors showed that frailty, defined by the LFI, was associated with the time to readmission.

It has to be acknowledged, however, that the discriminative ability of the LFI was only mediocre (AUC 0.66) in our study and is far from the results required for a reliable stand-alone test. Additionally, we only found an association between LFI as a metric variable and not frailty according to a cut-off of >4.5 in logistic regression analyses besides platelets and serum sodium. These findings emphasize that the risk of rehospitalization seems to be mainly determined by portal hypertension (as reflected by lower platelets) and poorer physical function (as reflected by LFI). In this context, the established cut-offs for frailty in cirrhotic outpatients (LFI > 4.5) may not apply for the prediction of rehospitalization. The ideal cut-off in our cohort was slightly higher at 4.62 according to the Youden’s index. However, our findings have to be interpreted in the context of the study design, and more reliable cut-offs should be developed in future larger multi-center studies.

Our current findings have several clinical implications. Using the LFI before discharge of patients with liver cirrhosis may help identify those in need for intensified support and interventions. There are several targets for improvement of frailty such as home-based physical therapy, pharmacologic therapies, as well as adequate pain management [16]. Additionally, nutritional interventions are critical not only to reduce the frequency of rehospitalizations but also to improve long-term prognosis [17]. In this context, our findings should also increase the awareness of the importance of clinical practice guidelines for nutrition in patients with liver cirrhosis [18,19]. In the future, experimental drugs to treat sarcopenia might also strengthen the therapeutic effect [20].

Several contextual limitations to our study have to be acknowledged. First, our study is based on data from a single tertiary care center, which may affect generalizability. Additionally, our sample size is only mediocre, and the results should be interpreted as a proof-of-concept. Therefore, larger multicenter studies are needed to establish robust cut-offs for predicting rehospitalization and to clarify whether the chair stands subtest is sufficient to predict rehospitalization or whether the full LFI is required. Second, due to our study design, we were only able to identify potential associations between different variables and short-term rehospitalization, and causality has to be proven in future studies. Last, we assessed the LFI only once during the last 48 h of each hospital stay according to clinical routine. Therefore, we are unable to determine the ideal timepoint for testing patients with the LFI to predict rehospitalization. Future studies should focus on longitudinal assessment of LFI during hospital stays of patients with liver cirrhosis.

In conclusion, our study adds to the growing body of evidence indicating the usefulness of the LFI for predicting clinical outcomes in patients with liver cirrhosis. We found that poorer results in the LFI were independently associated with a higher risk of 30-day rehospitalization in patients with liver cirrhosis, although the discriminative ability of the LFI was only mediocre. Nonetheless, our results warrant further research on this topic to validate our findings in larger cohorts and to establish robust cutoff values.

## Figures and Tables

**Table 1:** Baseline characteristics of the entire cohort at study inclusion and comparison between patients with and without rehospitalization within 30 days.

Variable		All Patients	Patients with Rehospitalization within 30 Days	Patients without Rehospitalization within 30 Days	*p* Value
Total, *n* (%)		*n* = 83 (100%)	*n* = 26 (31%)	*n* = 57 (69%)	
Age, y (IQR)		60 (51; 67)	60 (51; 69)	60 (50; 67)	0.36
Male gender, *n* (%)		50 (60%)	15 (57%)	35 (63%)	0.75
Charlson Comorbidity Index (IQR)		5 (3; 6)	5 (4; 7)	5 (3; 6)	0.50
Etiology	Alcohol, *n* (%)	55 (66%)	15 (57%)	40 (70%)	0.32
	Viral hepatitis, *n* (%)	7 (8%)	3 (12%)	4 (7%)	
	NAFLD, *n* (%)	9 (11%)	3 (12%)	6 (11%)	
	Cholestatic/Autoimmune, *n* (%)	3 (4%)	0 (0%)	3 (5%)	
	Other/mixed, *n* (%)	9 (11%)	5 (19%)	4 (7%)	
**Characteristics of liver cirrhosis**					
MELD score (IQR)		17 (13; 22)	21 (18; 24)	16 (11; 21)	**<0.01**
MELDNa score (IQR)		17 (13; 24)	22 (16; 27)	14 (12; 23)	**<0.01**
Child-Pugh A/B/C, *n* (%)		4/53/26 (5%/64%/31%)	0/16/10 (0%/62%/38%)	4/37/17 (7%/65%/28%)	0.29
History of ascites, *n* (%)		69 (83%)	25 (96%)	44 (75%)	**0.03**
History of HE, *n* (%)		29 (35%)	13 (50%)	16 (28%)	0.05
History of SBP, *n* (%)		17 (20%)	7 (27%)	10 (18%)	0.33
**Laboratory values**					
Sodium, mmol/L (IQR)		137 (133; 139)	133 (131; 138)	137 (134; 140)	**0.03**
Albumin, g/L (IQR)		28 (24; 32)	25 (23; 28)	29 (24; 33)	**0.04**
Bilirubin, mg/dL (IQR)		2.4 (1.4; 4.9)	3.2 (1.7; 8.2)	2.1 (1.4: 4.4)	0.96
WBC, nL (IQR)		5.2 (3.6; 7.8)	4.3 (3.1; 6.9)	5.8 (3.9; 8.1)	0.64
CRP, mg/L (IQR)		16 (5; 30)	18 (8; 33)	15 (5; 29)	0.38
Hemoglobin, g/dL (IQR)		10.1 (8.8; 12.1)	9.7 (8.2; 10.8)	10.2 (9.3; 12.3)	0.73
Platelets, nL (IQR)		88 (61; 142)	69 (52; 90)	103 (67; 156)	**<0.01**
**Liver Frailty Index (LFI)**					
LFI, (IQR)		4.5 (3.8; 5.1)	4.8 (4.2; 5.2)	4.4 (3.6; 4.9)	**0.02**
Frail, *n* (%)		43 (52%)	17 (65%)	26 (46%)	0.10
Dominant hand grip strength, kg (IQR)		22.6 (16.8; 29.8)	19.9 (16.7; 26.4)	23.1 (17.9; 30.7)	0.27
Chair stands, s (IQR)		18.1 (11.9; 28.3)	21.2 (16.5; 32.4)	15.7 (10.4; 24.4)	**0.02**
**Balance**					
Side (IQR)		10 (10; 10)			0.35
Semi-Tandem (IQR)		10 (5.4; 10)	9.8 (4.8; 10)	10 (5.8; 10)	0.12
Tandem (IQR)		5.9 (1.9; 10)	4.1 (0; 8.7)	6.7 (3.3; 10)	0.09

**Table 2:** Multivariable analyses of risk factors for 30-day rehospitalization in patients with cirrhosis.

Variable	Model 1 ^a^		Model 2 ^b^		Model 3 ^c^	
	OR	*p*	OR	*p*	OR	*p*
**Platelets (95% CI)**	0.98 (0.97–0.99)	<0.01	0.98 (0.96–0.99)	0.01	0.98 (0.96–0.99)	<0.01
**LFI** **(95% CI)**	2.36 (1.13–4.96)	0.02			2.36 (1.13–4.96)	0.02
**Sodium** **(95% CI)**			0.87 (0.77–0.98)	0.03		
**Harrell’s C-index**	0.78 (0.68–0.88)		0.78 (0.69–0.88)		0.78 (0.68–0.88)	

LFI, liver frailty index; CI, 95% confidence interval. Multivariable logistic regression model with a stepwise variable selection process (only the significant variables are displayed in the table). Variables that did not reach significance: ^a^ Hemoglobin (*p* = 0.11), sodium (*p* = 0.08), albumin (*p* = 0.29), history of OHE (*p* = 0.09), history of ascites (*p* = 0.10), MELD (*p* = 0.20), age (*p* = 0.22), gender (*p* = 0.44), Charlson Comorbidity Index (*p* = 0.95), ^b^ Hemoglobin (*p* = 0.10), MELD (*p* = 0.18), albumin (*p* = 0.50), history of OHE (*p* = 0.11), history of ascites (*p* = 0.11), age (*p* = 0.30), gender (*p* = 0.94), Charlson Comorbidity Index (*p* = 0.80), liver frailty index as a categorial variable (frail vs. pre-frail + robust) (*p* = 0.19), ^c^ Hemoglobin (*p* = 0.11), sodium (*p* = 0.08), albumin (*p* = 0.29), history of OHE (*p* = 0.09), history of ascites (*p* = 0.10), MELD (*p* = 0.20), age (*p* = 0.22), gender (*p* = 0.44), Charlson Comorbidity Index (*p* = 0.95), Child-Pugh Category (*p* = 0.81).

**Table 3:** Discriminative ability of the liver frailty index (LFI) and its subtest for prediction a rehospitalization within 30 days.

		Subtests of the LFI				
Variable	LFI	Chair Stands (s)	Tandem (s)	Semi-Tandem (s)	Side (s)	Hand Grip Strength (kg)
**AUC (95% CI)**	0.66 (0.54–0.78)	0.67 (0.55–0.79)	0.61 (0.48–0.75)	0.59 (0.46–0.73)	0.53 (0.40–0.67)	0.58 (0.44–0.71)
**Ideal cut-off**	4.62	19.23	5.74	9.88	9.84	21.89
**Sensitivity (95% CI)**	0.65 (0.44–0.82)	0.57 (0.37–0.76)	0.54 (0.34–0.73)	0.54 (0.33–0.73)	0.15 (0.05–0.36)	0.57 (0.37–0.76)
**Specificity (95% CI)**	0.65 (0.51–0.77)	0.58 (0.44–0.71)	0.54 (0.41–0.67)	0.66 (0.53–0.78)	0.91 (0.80–0.97)	0.58 (0.44–0.71)
**Positive Predictive Value (95% CI)**	0.46 (0.34–0.63)	0.38 (0.24–0.55)	0.35 (0.21–0.52)	0.42 (0.26–0.60)	0.44 (0.15–0.77)	0.38 (0.24–0.55)
**Negative Predictive Value (95% CI)**	0.80 (0.66–0.9)	0.75 (0.59–0.86)	0.72 (0.56–0.84)	0.76 (0.62–0.86)	0.70 (0.58–0.80)	0.75 (0.59–0.86)
**Positive likelihood ratio (95% CI)**	1.86 (1.19–2.92)	1.37 (0.88–2.14)	1.18 (0.74–1.86)	1.62 (0.97–2.69)	1.75 (0.51–6.00)	1.37 (0.88–2.14)
**Negative likelihood ratio (95% CI)**	0.53 (0.31–0.92)	0.73 (0.45–1.17)	0.84 (0.54–1.32)	0.69 (0.45–1.07)	0.93 (0.78–1.10)	0.73 (0.45–1.17)

AUC, area under the curve; LFI, liver frailty index; s, seconds; CI, 95% confidence interval.

## Data Availability

Raw data are available from the corresponding author upon reasonable request.

## Supplementary Materials

The following supporting information can be downloaded at: https://www.mdpi.com/article/10.3390/diagnostics12051069/s1, Table S1: Multivariable Cox-regression analyses of risk factors for 30-day rehospitalization in patients with cirrhosis.

## Abbreviations

AUC, area under the curve; CCM, Cirrhosis Centre Mainz; CI, confidence interval; CP, Child-Pugh; IQR, interquartile ranges; HR, hazard ratio; LFI, liver frailty index; MELD, model for end-stage liver disease; MELDNa, model for end-stage liver disease sodium score; NAFLD, non-alcoholic fatty liver disease; NASH, non-alcoholic steatohepatitis; NPV, negative predictive values; HE, hepatic encephalopathy; PPV, positive predictive values; ROC, receiver operating characteristic; SBP, spontaneous bacterial peritonitis; WBC, white blood cell count.
