# Supplementary material for: Liver Frailty Index for Prediction of Short-Term Rehospitalization in Patients with Liver Cirrhosis
Source: Diagnostics (Basel). 2022 Apr 24;12(5):1069. doi: 10.3390/diagnostics12051069 (PMC9139749; doi:10.3390/diagnostics12051069)
Supplement: Supplementary file 1 [file diagnostics-12-01069-s001.zip › diagnostics-1643102-supplementary.pdf]

**Supplementary Table S1.** Multivariable Cox-regression analyses of risk factors for 30-day rehospitalization in patients with cirrhosis.

| Variable | Model | |
|---|---|---|
| | **HR** | *p* |
| **Platelets (95% CI)** | 0.98 (0.97–0.99) | 0.01 |
| **LFI (95% CI)** | 1.65 (1.07–2.55) | 0.02 |

LFI, liver frailty index; CI, 95% confidence interval; Multivariable Cox-regression model with a stepwise variable selection process (only the significant variables are displayed in the table). Variables that did not reach significance: Haemoglobin ($p = 0.37$), sodium ($p = 0.08$), albumin ($p = 0.54$), history of OHE ($p = 0.25$), history of ascites ($p = 0.12$), MELD ($p = 0.26$), age ($p = 0.31$), gender ($p = 0.98$), Charlson Comorbidity Index ($p = 0.88$).
